# Une cholécystite lithiasique ectopique avec hernie de la ligne blanche: association rare révélée par inflammation pariétale abdominale

**DOI:** 10.11604/pamj.2021.38.83.27572

**Published:** 2021-01-25

**Authors:** Khalil Maamar, Mohammed Bouziane

**Affiliations:** 1 Université Mohammed VI, Chirurgie Générale A, Oujda, Maroc

**Keywords:** Cholécystite, hernie, ectopique, Cholecystitis, hernia, ectopic

## Abstract

Unlike anatomical variant, which is a functional adaptive deviation, congenital anomaly can cause pathological symptoms or, at least, it can pose clinical or paraclinical challenges. Ectopic gallbladder can be isolated or associated with abnormal liver development including lobe with hypertrophy, supernumerary lobe, or agenesis of hepatic lobe. Gallbladder usually lies in the right hypochondrium, below the lower quadrant of the right hepatic lobe. However, it can lie in other sites such as the right flank, the epigastrium, the periumbilical region, the right iliac fossa and even the left hypochondrium in patients with abdominal situs inversus. Its association with white-line hernia has never been reported; this poses clinical challenges and lead to difficulties in surgical approach. We here report a rare case of ectopic epigastric gallbladder lithiasis associated with white-line hernia in a 65-year-old patient admitted in the Emergency Room with diffuse abdominal pain. Abdominal examination showed epigastric swelling with inflammatory signs (Figure A). Radiological assessment revealed ectopic gallbladder lithiasis (Figure B, C). The patient underwent midline laparoscopic cholecystectomy. The anatomopathological examination of the surgical specimen confirmed the diagnosis.

## Image en médecine

À l'inverse de la variante anatomique qui constitue une déviation fonctionnellement adaptée, l'anomalie congénitale est susceptible d'avoir une expression pathologique ou, du moins, d'engendrer une problématique clinique ou paraclinique. L'existence d'ectopies vésiculaires peuvent être isolées ou associées à une anomalie du développement hépatique: hypertrophie d'un lobe, existence d'un lobe surnuméraire ou encore agénésie d'un lobe du foie. À côté de son siège habituel dans l'hypocondre droit, sous la face inférieure du lobe hépatique droit, la vésicule peut occuper d'autres sites comme le flanc droit, l'épigastre, la région péri-ombilicale, la fosse iliaque droite et même l'hypocondre gauche: cette dernière situation est même la règle en cas de situs inversus abdominal. L´association a une hernie de la ligne blanche n´as jamais été rapporté, et a engendré une problématique clinique et une difficulté d´abord chirurgicale. Nous rapportons un cas rare d´association de cholécystite lithiasique de siège ectopique épigastrique faisant hernie de la ligne blanche chez un patient de 65 ans admis aux urgences pour douleur abdominales diffuses, l´examen abdominal montre une tuméfaction épigastrique avec signe inflammatoire (A). Les explorations radiologiques ont révélé une cholécystite lithiasique de siège ectopique (B,C). Le patient a bénéficié d´une cholécystectomie par abord médiane. L´examen anatomopathologique de la pièce de résection a confirmé le diagnostic.

**Figure 1:**
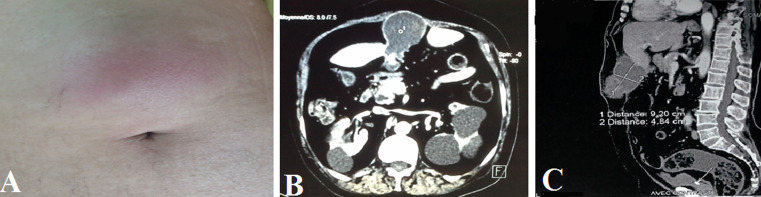
A) tuméfaction épigastrique avec signe inflammatoire; B,C) cholécystite lithiasique de siège ectopique

